# Optimization of extrusion conditions for the production of instant grain amaranth‐based porridge flour

**DOI:** 10.1002/fsn3.513

**Published:** 2017-09-12

**Authors:** Olamide A. Akande, Dorothy Nakimbugwe, Ivan M. Mukisa

**Affiliations:** ^1^ Department of Food Science and Technology Federal University of Technology Akure Ondo State Nigeria; ^2^ Department of Food Technology and Nutrition Makerere University Kampala Uganda

**Keywords:** Extrusion cooking, grain amaranth, instant porridge flour, optimization

## Abstract

Malnutrition is one of the foremost causes of death among children below 5 years in developing countries. Development of nutrient‐dense food formulations using locally available crops has been proposed as a means to combat this menace. This study optimized the extrusion process for the production of a nutritious amaranth‐based porridge flour. Least cost formulations containing grain amaranth, groundnut, iron‐rich beans, pumpkin, orange‐fleshed sweet potato, carrot, and maize were developed and evaluated by a sensory panel (*n* = 30) for acceptability using the 9‐point hedonic scale. Extrusion process of the most acceptable porridge flour was optimized by response surface methodology (RSM). Barrel temperature (130–170°C) and feed moisture content (14%–20%) were the independent variables which significantly (*p* < .05) affected in vitro protein digestibility, vitamin A retention, total polyphenol, phytic content, and iron and zinc extractabilities. Optimization of the extrusion process improved the nutritional quality of the instant flour.

## 1 INTRODUCTION

Malnutrition (protein–energy malnutrition and micronutrient deficiencies) is a severe problem facing developing countries and particularly affecting children under 5 years of age. Protein–energy malnutrition is associated with more than 50% of childhood mortality in developing countries (Thaoge et al., 2003). Twenty‐one percent of children have vitamin A deficiency and about 800,000 deaths in children are attributable to vitamin A deficiency globally (Tumwegamire et al., 2011). Globally, iron deficiency also affects about two billion people. About 800,000 deaths have been attributed to iron deficiency worldwide and 2.4% of global disability‐adjusted life years (DALYs) result from iron deficiency (Black, 2003). The most vulnerable group are children because of their high iron needs. According to Black (2003), 800,000 child deaths per year are attributable to zinc deficiency worldwide. In Uganda, 20% of children (under 5 years of age) are vitamin A deficient (FANTA‐2, 2010) and are at high risk of iron deficiency, as a result of low levels of iron in their diets (Tidemann‐Andersen, Acham, Maage, & Malde, 2011). In addition, 73% of children in Uganda are anemic (Tumwegamire et al., 2011). The prevalence of zinc deficiency in Uganda has been estimated to range between 20% and 69% in children (FANTA‐2, 2010). The high level of malnutrition in Uganda is partly attributed to the poor quality of diets (Muyonga, Nabakabya, Nakimbugwe, & Masinde, 2008; Tibagonzeka, Wambete, Muyinda, & Nakimbugwe, 2014). Causes of malnutrition can also be attributed to low nutrient density, low nutrient bioavailability, women's lack of time to prepare food frequently for young children, lack of fuel, and lack of a variety of foods to increase diet diversity (FANTA‐2, 2010). To tackle this problem of malnutrition, development of nutrient‐dense food formulations using locally available food materials have been advocated (Amegovu et al., 2014).

Malnutrition can be sustainably addressed through a cheap strategy of promoting consumption of nutritious foods or strategically blending locally available foods to produce nutrient‐dense products. In this study, grain amaranth, groundnuts, common dry beans, maize, orange‐fleshed sweet potato, carrots, and pumpkins were selected for inclusion in the formulation. The selected crops are readily available in Uganda and each contributes key nutrients of public health significance in Uganda, commonly lacking in the diets of vulnerable populations. Grain amaranth (*Amaranthus*) is a fast‐growing, high‐yielding, stress‐resistant, nutritious crop with potential to contribute to the alleviation of malnutrition and nutritional deficiencies (Muyonga et al., 2012). It contains superior quality protein with high lysine, an essential amino acid which is deficient in some cereals (such as maize). However, grain amaranth is deficient in β‐carotene (a precursor of vitamin A) (0 μg RAE/100 g or 2 IU/100 g) (USDA, 2014). Complementation with other food crops, whose cultivation and consumption are gaining popularity in East Africa, will enhance the nutritional quality of amaranth‐based porridges. Common dry beans are highly nutritious and a rich source of iron (8.2 mg/100 g) (USDA, 2014). Beans also contain more threonine and valine than the FAO/WHO reference protein (Muyonga et al., 2008). Beans have a commercial prospect and hold great potential for fighting hunger (Katungi et al., 2010). Groundnuts are a good source of energy (547 kcal/100 g) and zinc (3.27 mg/100 g) (USDA, 2014), and can help improve the levels of energy and zinc of the formulations. Maize is the world's most widely grown cereal, cultivated across a range of latitudes, altitudes, moisture regimes, slopes, and soil types (Murekatete, Fei, & Claver, 2010). Maize is mainly starchy and is a good source of energy (365 kcal/100 g), and also contains considerable amounts of iron and zinc (2.71 mg/100 g and 2.21 mg/100 g, respectively) (USDA, 2014). It has higher levels of leucine (13.75 g/100 g protein) than the FAO/WHO reference protein (7.0 g/100 g protein) (Muyonga et al., 2008) and is usually used for varying food preparations in Eastern Africa. Carrot, orange‐fleshed sweet potato, and pumpkin fruit are good sources of β‐carotene, a precursor of vitamin A, so they complement the β‐carotene deficiency of grain amaranth.

There has been a growing interest of using grain amaranth in combination with other plant sources to produce products of improved nutritional quality. In Uganda, the most common commercial use of grain amaranth is as a snack, while flour is the next common use (Kwikiriza et al., 2012). Products such as *baggia* (a cold extruded, deep fried, savory snack), *chapatti* (a thin pancake of unleavened bread cooked on a griddle), *kabalagala* (a flat‐rolled, deep‐fried snack made from ripe banana and cassava flour), porridges, and sauces were developed from grain amaranth composite flours in Uganda to address the dietary inadequacies (Ndagire, Muyonga, Manju, & Nakimbugwe, 2015; Tibagonzeka et al., 2014). Amaranth‐based products have been developed employing processes such as popping, frying, steaming, cooking, and extrusion.

Extrusion cooking is a feasible alternative for manufacturing water reconstitutable foods for blended flours (Pathania, Singh, Sharma, Sharma, & Singla, 2013). It is a high‐temperature/short‐time technology that offers numerous advantages including versatility, high productivity, low operating costs, energy efficiency, and high quality of resulting products (Milán‐Carrillo, Montoya‐Rodríguez, Gutiérrez‐Dorado, Perales‐Sánchez, & Reyes‐Moreno, 2012). Extrusion cooking is characterized by its capacity of increasing the digestibility of starch and protein (Diaz et al., 2013). Extrusion also breaks down mineral–antinutrients complexes by hydrolysis, thereby increasing the mineral availability in the extrudates, altering vitamins, and eliminating antinutrient factors such as phytic acid, polyphenols which modifies the nutritional properties of the extrudates (Singh, Gamlath, & Wakeling, 2007; Sundarrajan, 2014). The actual extrusion conditions required to ensure appropriate nutritional and sensory quality depends on the food ingredients. Various studies have been carried out on optimizing the effect of extrusion conditions particularly on physical characteristics (Omwamba & Mahungu 2014), physicochemical properties (Durgadevi & Nazni, 2012; Ndagire et al., 2015; Nkundabombi, Nakimbugwe, & Muyonga, 2016), and functional properties (Bhise, Kaur, Manikantan, & Singh, 2013; Filli, Nkama, Abubakar, & Jideani, 2010; Pathania et al., 2013). However, information on optimizing the effects of extrusion on high β‐carotene, iron, and zinc composite foods is limited. This study therefore determined the effect of incorporating different ingredients and preprocessing on product acceptability and optimized the extrusion conditions (barrel temperature and feed moisture) of amaranth‐based flours to maximize β‐carotene (precursor of vitamin A) retention, increase in in vitro protein digestibility, iron and zinc extractability, and reduce total polyphenol and phytic contents.

## 2 MATERIALS AND METHODS

Affordable and locally and readily available foods (groundnut, iron‐enriched ROBA 1 beans, pumpkin, orange‐fleshed sweet potatoes, carrots, and maize) that complement the nutritional profile of grain amaranth were selected on the basis that they are rich sources of the target nutrients (protein, energy, iron, zinc, and β‐carotene [precursor of vitamin A]).

### 2.1 Raw materials

Grain amaranth, common bean (ROBA 1 variety), and maize flours were obtained from Nutreal Ltd., Kampala, Uganda. Raw orange‐fleshed sweet potato tubers (NASPOT‐10 variety) were obtained from a farmer in Bombo, Uganda. Fresh carrots, pumpkin, and groundnut were purchased from Kalerwe market, Kampala, Uganda.

### 2.2 Preparation of flours

Groundnuts were sorted and milled into flour using a local cast iron mill. The method of Dauthy (1995) was adopted for the processing of the orange‐fleshed sweet potatoes and carrot flour. Pumpkins were processed following the method of Pongjanta, Naulbunrang, Kawngdang, Manon, and Thepjaikat (2006).

### 2.3 Product development

#### 2.3.1 Formulations

Concept 4 creative software (Creative Formulation Concepts, LLC, Annapolis, MD, USA) was used to generate six formulations (Table 1) whose composite contributes 90% protein, 30% energy, 45% vitamin A, 60% iron, and 70% zinc of the recommended dietary intake of children below 5 years based on the guidelines by the Institute of Medicine of the National Academics (2006).

**Table 1:** The amaranth‐based formulations designed to meet 90% protein, 30% energy, 45% vitamin A, 60% iron, and 70% zinc of the recommended dietary intake of children below 5 years of age

Formulations	Proportion (%) of the different ingredients in the composite flour						
	Grain amaranth	ROBA beans	Groundnut	Carrot	OFSP	Pumpkin	Maize
1	40	20	15	15	0	0	10
2	40	20	15	0	15	0	10
3	35	20	15	0	0	20	10
4	45	20	15	0	10	0	10
5	50	20	15	5	0	0	10
6	40	20	15	0	0	15	10

### 2.4 Screening of formulations for acceptability

The formulations were prepared and conditioned to a moisture content of 20% and thoroughly mixed using a mixer for 15 min and extruded using a DP70‐III double screw inflating food machine (Jinan Eagle Machine Co. Ltd., Jinan, China). The extruder conditions were feeding frequency of 30 Hz, cutting frequency of 50 Hz, and barrel temperature of 60°C, 130°C, and 150°C in first, second, and third zones, respectively. After extrusion, the samples were cooled to room temperature under natural convection conditions. The samples were then milled into flour using a 30 B‐C milling machine (Changzhou Erbang Drying Equipment Co. Ltd., China), packed in polythene bags, and stored at 4°C.

Six different porridges were prepared by mixing 200 g of each of the composite flours in 800 ml of boiling water with constant stirring for about 4 min. Thirty grams of sugar was added per liter of the ready porridges. Sensory acceptability was determined by a semitrained panel (*n* = 30) mainly comprised of students in the School of Food Technology, Nutrition, and Bio‐engineering, Makerere University. A 9‐point hedonic scale was used to score the acceptability of the different porridges.

### 2.5 Optimization of extrusion conditions for the most acceptable instant flour

After sensory acceptability screening, the extrusion process of the most acceptable formulation was optimized. Each independent variable (barrel temperature, *X*_1_ and feed moisture, *X*_2_) was varied as shown in Table 2. The dependent variables were protein content and digestibility, iron extractability, zinc extractability, vitamin A, phytate, and polyphenol contents.

**Table 2:** Independent variables and levels for extrusion used in the central composite design

Independent variables	Symbol	Coded variables		
		−1	0	1
Barrel temperature (third zone) (°C)	*X*_1_	130	150	170
Feed moisture (%)	*X*_2_	14	17	20

The dependent variables were expressed individually as a function of the independent variables (Table 2). Data were fitted to a second‐order approximation model using Equation (1).

(1) $$Y=B_{O}+\sum_{i-1}^{k}B_{i}X_{i}+\sum_{i-1}^{k}B_{ii}X_{i}^{2}+\sum_{\begin{array}{c} i-1\\ i<j \end{array}}^{k}B_{ij}X_{i}X_{j+\varepsilon }$$

where *Y* is the response function, *X*_i_ is the feed moisture content, *X*_j_ is the barrel temperature, ε is the random error, *B*_o_ the center point of the system, *B*_i_, *B*_ii_, and *B*_ij_ represent the coefficients of the linear, quadratic, and interactive effects of the dependent variables, respectively, and *X*_i_, *X*_i_^2^, and *X*_i_ *X*_j_ represent the linear, quadratic, and interactive effects, respectively, of the independent variable.

The desirability function approach (DFA) was used to simultaneously optimize the amaranth‐based flour's in vitro protein digestibility, total polyphenol, phytate, protein content, vitamin A retention, and iron and zinc extractability after carrying out the analyses below.

#### 2.5.1 Physicochemical analyses

Total phytate content was determined by the titrimetric method described by Sarkiyayi and Agar (2010), with the following slight modifications (the extract collected was decolorized using 5 g of activated charcoal powder, the filtrate [10 ml] was added to 100 ml conical flask containing 5 ml of 0.3% ammonium thiocyanate, and the mixture was then titrated with standard ammonium ferrous sulfate to a golden yellow color end point). Total extractable phenolics were determined according to the method of Julkunen (1985). Iron and zinc contents were determined using the Savant atomic absorption spectrophotometer (GBC Scientific Equipment Pty Ltd., Dandenong, Victoria, Australia, Model: A.C.N 005472686) following the method described by Okalebo, Gathua, and &Woomer (2002). Iron and zinc extractability were determined using a method by Duhan, Khetarpaul, and Bishnoi (2002). Beta‐carotenoid content was quantified using the method described by Bechoff et al. (2011). In vitro protein digestibility was determined using pepsin–pancreatin enzyme system (Chavan, McKenzie, & Shahidi, 2001; Saunders, Connor, Booth, Bickoff, & Kohler, 1973). Protein content of the sample was determined using the Kjeldahl method (AOAC 1990) before and after digestion and digestibility was calculated using the formula:

(2) $$\%\;\text{in vitro}\;\text{protein digestibility}=\frac{\left(A-B\right)}{A}$$

where A is % protein in the sample before digestion and B is % protein after enzyme digestion.

#### 2.5.2 Sensory analysis

After optimization, consumer acceptability tests were carried out by 48 semitrained panelists using a 9‐point hedonic scale (1 = dislike extremely to 9 = like extremely). The instant porridge was prepared as described earlier (*Screening of formulations for acceptability*). About 10 ml of each sample was presented to panelists in identical white containers, coded with three‐digit random numbers. Serving order was randomized for each panelist in individual sensory booths. Commercial bottled water was provided to rinse the mouth before and between tasting samples. Panelists were asked to score the acceptability for color, taste, flavor, mouth feel, consistency, and overall acceptability. Acceptability of the amaranth‐based porridge was compared with a commercial maize‐based instant porridge commonly consumed in Kampala city, Uganda.

### 2.6 Characteristics of porridge made from the optimized flour

#### 2.6.1 Proximate analysis

The analyses below were carried out on the instant porridge produced under optimum conditions.

Moisture content was determined by oven drying overnight at 98°C (AOAC (Association of Official Analytical Chemists), 1999). Crude protein content was determined using Kjedahl method (AOAC, 1990), ash content by igniting a dried, ground sample in a furnace at 550°C (AOAC, 1990), fat content was determined using the Soxtec apparatus and total carbohydrate was calculated by difference (AOAC, 1990), dietary fiber was determined using the FIBERTEC, and gross energy using the bomb calorimetry method (AOAC, 1999).

##### Nutrient density determination

Porridges were prepared with different concentrations of flour (15%, 18%, 19%, and 20%) and their viscosities measured using a Brookfield DV II + Pro Viscometer at 55°C. Flour rates that could produce porridges of drinkable viscosities (2,500–3,000 cP) were suitable for child feeding (Mosha & Svanberg, 1983). Energy, protein, iron, zinc, and vitamin A densities of the porridges with the desired viscosity (2,500–3,000 cP) were calculated.

(3) $$\text{Nutrient}\;\text{density}\;(100\;\text{ml}{}^{-1})=\text{Flour}\;\text{rate}/100\;\text{ml}\times \text{Nutrient}/100\;g$$

#### 2.6.2 Determination of pasting properties of instant porridges

Pasting properties of the instant porridges were determined using a Rapid Visco Analyser (RVA‐4, Newport Scientific). The pasting temperature (PT), peak viscosity (PV, the maximum hot paste viscosity), holding strength or trough viscosity (the trough at the minimum hot paste viscosity), final viscosity, breakdown (BD, peak viscosity‐holding strength or trough viscosity), and setback (SB, final viscosity‐holding strength) were obtained with Thermocline for Windows software. The viscosities were presented in centipoise (cP).

### 2.7 Statistical data analysis

Data for optimizing amaranth‐based flour extrusion conditions was analyzed by response surface methodology (RSM) procedures using design‐expert statistical software (DX 6.0; Stat‐Ease, Inc., Minneapolis, MN, USA). Data for consumer acceptability and pasting properties were analyzed using the Statistical Package for Social Science (SPSS) software (version 17). Mean and standard deviations were computed. T test and analysis of variance (ANOVA) were used to calculate significant differences in treatment means and least significant difference technique was used for separation of means (alpha level of 0.05).

## 3 RESULTS AND DISCUSSION

### 3.1 Screening of formulations for acceptability

Formulations of the composite flours significantly (*p* ≤ .05) affected the consumer acceptability scores of porridge (Table 3). The formulation with grain amaranth, beans, groundnut, maize, and dried pumpkin flour (Formulation 6) was significantly (*p* ≤ .05) different and more acceptable than other formulations. Formulation with grain amaranth, beans, groundnut, maize, and fresh potato flour (Formulation 1) had the lowest score. Therefore, formulation 6 was selected for optimization. Acceptability of this formulation could be attributed to the inclusion of pumpkin flour which improved the textural quality and flavor characteristics of the porridge thus masking the taste of grain amaranth. Kulkarni and Joshi (2013) also reported an increase in acceptability with increase in replacement of gram flour with pumpkin flour in a developed *bhajjiya* (an Indian savory).

**Table 3:** Consumer acceptability scores of porridges prepared from different formulations of amaranth‐based composite flours

Formulation	Color	Aroma	Texture	Thickness	Taste	After taste	Overall appearance	Overall acceptability
1	5.8 ± 1.6^cd^	6.4 ± 1.1^b^	7.0 ± 1.0^ab^	6.9 ± 1.1^ab^	6.9 ± 1.0^ab^	6.7 ± 1.0^a^	6.7 ± 0.8^b^	7.1 ± 0.8^bc^
2	6.4 ± 1.6^abc^	6.1 ± 1.3^bc^	6.6 ± 1.2^b^	6.2 ± 1.7^bc^	5.9 ± 1.6^c^	6.4 ± 1.5^a^	6.8 ± 1.2^b^	6.7 ± 1.1^c^
3	5.2 ± 2.0^d^	5.5 ± 1.8^c^	5.2 ± 1.6^c^	5.5 ± 1.7^c^	4.6 ± 1.6^d^	5.2 ± 1.8^b^	5.1 ± 1.4^c^	5.4 ± 1.0^d^
4	6.2 ± 1.4^bc^	6.3 ± 1.3^bc^	5.9 ± 1.7^c^	6.6 ± 1.6^ab^	6.5 ± 1.4^bc^	6.5 ± 1.4^a^	6.5 ± 1.0^b^	6.8 ± 0.9^c^
5	7.3 ± 1.4^a^	6.1 ± 1.4^bc^	6.8 ± 1.1^ab^	7.2 ± 0.9^a^	6.6 ± 1.4^bc^	6.7 ± 1.0^a^	7.6 ± 0.9^a^	7.4 ± 0.7^b^
6	7.1 ± 1.7^ab^	7.4 ± 1.0^a^	7.5 ± 1.0^a^	6.7 ± 1.6^ab^	7.4 ± 1.0^a^	6.9 ± 1.3^a^	7.7 ± 1.0^a^	7.9 ± 0.7^a^

Values show mean ± SD (*n* = 30). Figures in the same column with the same superscript are not significantly (*p* > .05) different. A 9‐point hedonic scale was used with 1 = dislike extremely, 5 = neither like nor dislike, and 9 = like extremely. 1 = Grain amaranth (40%), ROBA beans (20%), groundnut (15%), maize (10%), and fresh OFSP (15%). 2 = Grain amaranth (40%), ROBA beans (20%), groundnut (15%), maize (10%), and fresh carrot (15%). 3 = Grain amaranth (35%), ROBA beans (20%), groundnut (15%), maize (10%), and fresh pumpkin (20%). 4 = Grain amaranth (45%), ROBA beans (20%), groundnut (15%), maize (10%), and OFSP flour (10%). 5 = Grain amaranth (50%), ROBA beans (20%), groundnut (15%), maize (10%), and carrot flour (5%). 6 = Grain amaranth (40%), ROBA beans (20%), groundnut (15%), maize (10%), and pumpkin flour (15%).

### 3.2 Effect of extrusion conditions on nutritional quality of the composite flours

Extrusion conditions (feed moisture content and barrel temperature) affected the nutritional composition of the instant amaranth‐based flours (Table 4). The relationships between feed moisture content and barrel temperature were represented by a number of predictive models. The negative coefficients of the linear terms of moisture and temperature in the predictive equations indicate that the response decreases with increase in the variables, while the positive coefficients indicate that responses increase with increase in feed moisture content and barrel temperature.

**Table 4:** Effect of extrusion cooking on nutritional quality of the amaranth‐based flour

Runs	FMC (%)	BT (°C)	FMC (%)	BT (°C)	PC (%)	PD (%)	Vit. A (μg RAE/100 g)	PP mg/100 g GAE	PA mg/100 g	Zn (%)	Fe (%)
1	−1	−1	14	130	18.14	85.23	590.80	261.25	69.61	43.38	15.12
2	−1	−1	14	130	18.16	87.36	589.65	257.26	69.25	43.30	15.55
3	0	−1	17	130	18.29	80.53	542.14	247.53	60.54	41.38	15.99
4	−1	0	20	130	18.31	84.91	501.13	245.86	68.53	42.68	17.01
5	0	0	14	150	17.92	86.87	589.21	215.37	63.28	42.11	17.33
6	0	0	17	150	18.01	81.02	547.63	208.84	53.85	45.17	16.46
7	1	0	17	150	17.99	81.65	522.06	208.18	55.93	45.68	16.62
8	‐1	1	20	150	17.81	87.44	550.67	193.15	57.12	46.58	15.77
9	‐1	1	14	170	18.35	81.76	551.55	219.53	54.33	49.53	17.26
10	0	1	17	170	18.16	80.00	596.00	214.71	54.96	48.45	15.60
11	1	1	20	170	18.08	80.35	539.26	214.96	55.69	48.61	17.18
12	1	1	20	170	18.13	79.17	544.43	214.60	56.47	48.29	17.40
Raw flour	–	–	–	–	18.35	77.69	693.85	268.67	78.05	29.05	7.10

FMC, feed moisture content; BT, barrel temperature; PC, protein content; PD, protein digestibility; Vit. A, Vitamin; PP, total polyphenol content; PA, phytic acid; Zn, zinc extractability; Fe, iron extractability.

The predictive models were tested for adequacy and fitness by analyses of variance (ANOVA). According to Myers and Montgomery (2002), a good predictive model should have an adjusted *R* ^2^ (coefficient of determination) ≥ 0.80, a significance level of *p* < .05, and coefficients of variance and (CV) values ≤ 10%; all these parameters could be used to decide the satisfaction of the modeling.

#### 3.2.1 Protein content

Equation (4) describes the apparent effect of feed moisture content and barrel temperature on protein content.

(4) $$\begin{array}{cc} \mathrm{Protein}\;\mathrm{content}=\; & 18.00-0.065X_{1}-0.055X_{2}-0.10X_{1}X_{2}\\ & +\;0.22X_{1}^{2}-0.13X_{2}^{2}+0.034X_{1}^{2}X_{2}\\ & +\;0.064X_{1}X_{2}^{2}+0.14X_{1}^{2}X_{2}^{2}\;\left(p=.0028,\;R^{2}=0.9943\right) \end{array}$$

Barrel temperature (*X*_1_) and feed moisture content (*X*_2_) had a negative linear and interactive effect on protein content of the instant amaranth‐based flour (Equation (4)). Increase in temperature caused a significant reduction in protein content. The losses in protein could be attributed to protein denaturation and apparent partial loss of certain amino acids along with other nitrogenous compounds on heating (Rehman & Shah, 2005). High temperatures favor Maillard reactions leading to degradation of amino acids such as lysine which is easily degraded because of its two available reactive amino groups (Singh et al., 2007).

#### 3.2.2 In vitro protein digestibility

(5) $$\begin{array}{cc} \mathrm{Protein}\;\mathrm{digestibility}=\; & 82.20-1.92X_{1}-0.69X_{2}-0.26X_{1}X_{2}-2.79X_{1}^{2}\\ & +\;4.10X_{2}^{2}\;\left(p=.0240,\;R^{2}=0.8354\right) \end{array}$$

The quadratic effect of feed moisture (*X*_2_) on in vitro protein digestibility resulted in an increase in in vitro protein digestibility (Equation (5)). Negative interactive effects existed between barrel temperature and feed moisture content. The combination of feed moisture content of 20% and barrel temperature of 150°C resulted in the highest in vitro protein digestibility (Table 4). Protein nutritional value is dependent on the quantity, availability and digestibility of essential amino acids. Digestibility is considered as the most important determinant of protein quality (FAO/WHO/UNU, 1985). In vitro protein digestibility (IVPD) has been reported to closely relate to true digestibility, and is normally used as a quick and convenient alternative to in vivo protein digestibility (Adam, Hua, Chamba, & Gasmalla, 2013). The increased IVPD of the instant amaranth‐based flour produced at 20% feed moisture content and 150°C barrel temperature indicates that the protein may be more available for the body's nourishment than that of the products produced at different feed moisture content and barrel temperature combinations (Adam et al., 2013). According to Muyonga, Andabati, and Ssepuuya (2014), the nature of the change in protein digestibility resulting from heat treatment seems to relate partly to the extent of formation of complexes between proteins and other grain components and the level of matrix disintegration, which impacts the access of proteolytic enzymes to protein bodies. Reduction in polyphenols and phytic acid could also be responsible for the observed increase in protein digestibility (Equations (7) and (8)).

#### 3.2.3 Vitamin A

Barrel temperature (*X*_1_) had negative linear effect on vitamin A content implying that it decreases with increasing barrel temperature (Equation (6)). Feed moisture content (*X*_2_) had positive linear and quadratic effects. Loss in vitamin A may be attributed to thermal degradation which appears to be the major factor contributing to β‐carotene loss during extrusion (Singh et al., 2007). The relationship between vitamin A content, barrel temperature, and feed moisture content was significant (*p* < .05).

(6) $$\begin{array}{cc} \mathrm{Vitamin}\;A=\; & 534.85-26.93X_{1}+19.27X_{2}+19.85X_{1}X_{2}+34.23X_{1}^{2}\\ & +\;35.10X_{2}^{2}-5.43X_{1}^{2}X_{2}-26.42X_{1}X_{2}^{2}-57.98X_{1}^{2}\;\\ & \left(p=.0392,\;R^{2}=0.9657\right) \end{array}$$

#### 3.2.4 Total polyphenol

Barrel temperature (*X*_1_) and feed moisture content (*X*_2_) had negative linear effects on the total polyphenol content of the composite flour (Equation (7)).

(7) $$\begin{array}{cc} \mathrm{Total}\;\mathrm{polyphenol}=\; & 208.51-16.41X_{1}-6.11X_{2}+2.16X_{1}X_{2}\\ \; & +\;22.61X_{1}^{2}-9.25X_{2}^{2}-1.57X_{1}^{2}X_{2}-1.29X_{1}X_{2}^{2}\\ \; & +\;12.99X_{1}^{2}X_{2}^{2}\;\left(p=.0004,\;R^{2}=0.9985\right) \end{array}$$

Total polyphenol content of the instant flours reduced with increasing feed moisture content and barrel temperature. Polyphenols are naturally occurring substances in plants which inhibit non‐heme‐iron absorption and interfere with protein digestibility (Bravo, 1998) by inhibiting proteolytic enzymes (ElShazali, Nahid, Salma, Isam, & Elfadil, 2011). Polyphenols have the ability to form complexes with iron and other cations through their carboxylic and hydroxylic groups, and thus interfere with the intestinal absorption of minerals (Bravo, 1998). This makes polyphenols undesirable in foods. Extrusion variables (barrel temperature and feed moisture content) caused a significant reduction of polyphenols. According to Singh et al. (2007), it might be possible that lost phenolics reacted with themselves or with other compounds to form larger insoluble materials.

#### 3.2.5 Phytic acid

Equation (8) defines the effect of extrusion cooking on the phytic acid content of the instant composite flour.

(8) $$\begin{array}{cc} \mathrm{Phytic}\;\mathrm{acid}\;\mathrm{content}=\; & 55.18-2.79X_{1}-3.08X_{2}\\ \; & +\;0.63X_{1}X_{2}+2.29X_{1}^{2}-4.74X_{2}^{2}-3.29X_{1}^{2}X_{2}\\ \; & -4.10X_{1}X_{2}^{2}\;\left(p=.0004,\;R^{2}=0.9925\right) \end{array}$$

The barrel temperature (*X*_1_) and feed moisture content (*X*_2_) had significant negative linear effects on the phytic acid content of the flours. Phytic acid is present in foods in varying concentrations of 0.1%–6.0% (Fereidoon, 1997). Its structure is highly negatively charged, making it a very reactive compound that attracts positively charged ions such as those of iron, zinc making them unavailable for absorption and utilization by the body (Ramakrishna, Jhansi, & Ramakrishna, 2006). Phytic acid can also react with charged groups of proteins, either directly or indirectly. Phytic acid interacts with starch molecules, directly via hydrogen bonding, with phosphate groups or indirectly through proteins to which it is attached (Fereidoon, 1997). Such bindings reduce the solubility and digestibility of protein and starch components of food (Fereidoon, 1997). Phytic acid also forms complexes with proteins and proteases of the intestinal tract, inhibiting digestion. Increasing barrel temperature and feed moisture content caused a significant reduction in phytic acid content of the amaranth‐based instant flour (Table 4). The observed reduction in phytic content in foods during heat treatments such as extrusion may be partly due to the heat labile nature of phytic acid and the formation of insoluble complexes between phytate and other components (Kaur, Savita, Baljit, & Dar, 2013).

#### 3.2.6 Zinc extractability

The predictive model describing the effects of barrel temperature and feed moisture content on zinc extractability of the amaranth‐based instant flour was significant (*p* < .05). Barrel temperature (*X*_1_) and feed moisture content (*X*_2_) had positive linear effects on zinc extractability (Equation (9)). Extractable minerals in a food are those which are soluble in 0.03 N hydrochloric acid (HCl), which is the concentration of HCl found in the human stomach (Anjum et al., 2012). Extractability of minerals is an index of their bioavailability (Duhan et al., 2002). Extrusion cooking resulted in increase of the zinc extractability of the amaranth‐based instant flours. Similar results were reported by Alonso, Rubio, Muzquiz, and Marzo (2001). Increase in zinc extractability can be attributed to hydrolysis of phytate to release phosphate molecules, partly attributed to the destruction of polyphenols and reorganization of dietary fiber components changing their chelating properties by extrusion cooking (Singh et al., 2007). Indeed results of the current study also show that phytate and polyphenols decrease with increasing temperature and moisture content (Equation (7) and (8)).

(9) $$\begin{array}{cc} \mathrm{Zinc}\;\mathrm{extractability}=\; & 45.43+3.53X_{1}+2.24X_{2}-0.10X_{1}X_{2}-0.51X_{1}^{2}\\ \; & -\;1.08X_{2}^{2}-2.68X_{1}^{2}X_{2}-0.55X_{1}X_{2}^{2}\\ \; & -\;2.17X_{1}^{2}X_{2}^{2}\;\left(p=.0006,\;R^{2}=0.9979\right) \end{array}$$

#### 3.2.7 Iron extractability

Equation (10) shows the effect of barrel temperature (*X*_1_) and feed moisture content (*X*_2_) on the iron extractability of the extruded flours.

(10) $$\begin{array}{cc} \mathrm{Iron}\;\mathrm{extractability}=\; & 16.54-0.20X_{1}-0.78X_{2}-0.41X_{1}X_{2}-0.75X_{1}^{2}\\ \; & +\;7.00X_{2}^{2}+1.21X_{1}^{2}X_{2}+0.76X_{2}^{2}\\ \; & +\;0.92X_{1}^{2}X_{2}^{2}\;\left(p=.0160,\;R^{2}=0.9815\right) \end{array}$$

Feed moisture content had a positive quadratic effect (Equation (10)) on iron extractability. Iron extractability increased with increase in feed moisture content at barrel temperature of 130°C but decreased with increase in feed moisture content at barrel temperature of 150°C (Table 4). The effect of feed moisture content at 170°C were not clear as no trend could be established (Table 4). Increase in iron availability during extrusion cooking is usually attributed to improvement in its absorption by reducing other factors, such as phytate, that inhibit absorption (Alonso et al., 2001). From the results of this study, phytate decreased with increase in feed moisture content and extrusion temperature (Equation (8)).

### 3.3 Optimum processing conditions

A barrel temperature of 169°C and feed moisture content of 14% were chosen as the optimum conditions since they had the highest desirability (0.756). These processing conditions resulted in instant amaranth‐based flour with 18.32% protein content, 82.82% protein digestibility, 217.63 mg/100 g total polyphenol content, 54.86 mg/100 g total phytates, 553. 80 μg/RAE 100 g vitamin A content, 49.06% extractable zinc, and 17.32% extractable iron.

### 3.4 Consumer acceptability of the optimized amaranth‐based porridge

The consumer acceptability of the amaranth‐based composite instant porridge was compared with a common commercial maize‐based instant porridge (Table 5). The mean acceptability scores for all attributes ranged from 6.12 to 7.76 for the amaranth‐based porridge and 6.19–7.19 for the commercial maize‐based instant porridge. Amaranth‐based porridge and the control porridge received similar scores on all attributes except texture, thickness, and aftertaste. The acceptability scores for thickness and texture of the amaranth‐based porridge were significantly (*p* < .05) higher than for the control porridge. The mean acceptability score for aftertaste of the control porridge was significantly (*p* < .05) higher than that for the amaranth‐based porridge. Inclusion of pumpkin flour in the amaranth‐based instant porridge resulted in a product with a very smooth consistency which is most likely responsible for higher texture and thickness scores. From the panelists' point of view, amaranth‐based porridge leaves an undesirable distinct aftertaste when consumed unlike the maize‐based porridge.

**Table 5:** Comparison of sensory acceptability scores for amaranth‐based porridge and a commercial maize‐based porridge

Attributes	Amaranth‐based composite porridge	Maize‐based porridge
Color	7.29 ± 1.31^a^	7.19 ± 1.32^a^
Aroma	6.58 ± 1.42^a^	6.90 ± 1.37^a^
Texture	7.53 ± 1.01^a^	6.19 ± 1.69^b^
Thickness	7.76 ± 0.90^a^	6.24 ± 1.73^b^
Taste	6.82 ± 1.59^a^	6.71 ± 1.68^a^
After taste	6.12 ± 1.80^b^	7.09 ± 1.22^a^
Overall appearance	7.18 ± 0.88^a^	6.57 ± 1.33^a^
Overall acceptability	6.94 ± 1.09^a^	6.81 ± 1.21^a^

Values show mean ± SD (*n* = 48) at 5%. Figures in the same row with the same superscript are not significantly different (*p* > .05).

### 3.5 Physical characteristics of porridge made from the optimized amaranth‐based flour

#### 3.5.1 Viscosity analysis of the amaranth‐based and maize‐based instant porridges

**Figure 1:**
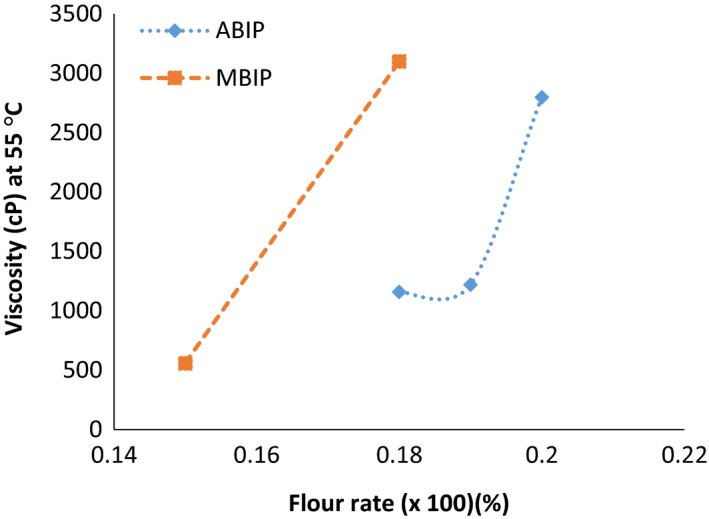
Viscosities (cP) of the amaranth‐based instant porridge and maize‐based instant porridges at varied flour rates. ABIP, amaranth‐based instant porridge; MBIP, maize‐based instant porridge

The amaranth‐based and control instant porridges attained the drinking viscosity (2,500–3,000 cP) at 20% (20 g/100 ml) and 18% (18 g/100 ml) flour rates, respectively (Figure 1). The higher flour rate of the amaranth‐based porridge could be attributed to higher starch damage evident by the result of pasting properties (Table 7). This flour rate gave reconstituted product appropriate for infant feeding. Viscosity is important in food intake because it contributes to an increase or decrease in the bulk of a cooked cereal product and affects taste intensity (Mburu, Gikonyo, Kenji, & Mwasaru, 2011).

#### 3.5.2 Nutrient density of the amaranth‐based porridge

The energy, iron, and vitamin A densities of the amaranth‐based instant porridge were higher than that of the maize‐based instant porridge (Table 6). However, protein and zinc densities of the amaranth‐based instant porridge were lower than that of the commercial maize‐based instant porridge.

**Table 6:** Calculated nutrient density (per 100 ml) of the porridges

Nutrient	Amaranth‐based instant porridge (20 g/100 ml)	Maize‐based instant porridge (18 g/100 ml)
Energy (kcal)	90.75	75.73
Protein (g)	3.79	4.32
Iron (mg)	2.40	1.44
Zinc (mg)	0.69	0.90
Vitamin A (μgRAE)	100.00	14.98

The high energy, iron, and vitamin A densities of amaranth‐based instant porridge (Table 6) when compared to maize‐based instant porridge can be attributed to nutrient complementation especially for the vitamin A density. The presence of soya beans flour in the commercial maize‐based instant porridge could have accounted for its higher protein and zinc densities. Soya bean is known to be a rich source of protein (36.49%) and zinc (4.89%) (USDA, 2014). The high nutrient densities of the amaranth‐based instant porridge show that it is suitable for infant and young children feeding and can help reduce protein–energy malnutrition and micronutrients (iron, zinc, and vitamin A) deficiencies.

### 3.6 Pasting properties of the amaranth‐based porridge and a commercial control

The pasting properties of amaranth‐based instant porridge are presented in Table 7. Amaranth‐based instant porridge had a significantly (*p* < .05) higher breakdown viscosity, peak time, and pasting temperature, and a significantly (*p* < .05) lower peak viscosity, trough I, and final viscosity when compared to the commercial maize‐based instant porridge.

**Table 7:** Comparison of the pasting properties of amaranth‐based porridge and a commercial‐based porridge

Pasting properties	Amaranth‐based instant porridge	Commercial porridge
Peak viscosity (cP)	12.50 ± 0.08^b^	13.46 ± 0.21^a^
Trough I (cP)	12.04 ± 0.04^b^	13.29 ± 0.21^a^
Breakdown viscosity (cP)	0.46 ± 0.04^a^	0.17 ± 0.00^b^
Final viscosity (cP)	20.13 ± 0.05^b^	21.29 ± 0.54^a^
Setback I (cP)	8.08 ± 0.09	8.00 ± 0.33
Setback II (cP)	7.62 ± 0.13	7.83 ± 0.34
Peak time (min)	6.83 ± 0.17^a^	5.60 ± 0.07^b^
Pasting temperature (°C)	94.75 ± 0.00^a^	72.88 ± 0.23^b^

Values are means ± SD (*n* = 2). Values in the same row with different superscripts are significantly (*p* ≤ .05) different.

Pasting properties are important indices for determining the cooking quality of flours (PBIP, 1995). Peak viscosity was not very pronounced for porridges produced from the amaranth‐based instant flour. Low viscosity is an indication of molecular and structural degradation in the starch granules during extrusion cooking (Ilo, Liu, & Berghofer, 1999). Some previous studies have also reported this similar results (Ilo et al., 1999; Menegassi, Pilosof, & Arêas, 2011). Amaranth‐based instant porridge had lower peak viscosity than commercial maize‐based instant porridge implying that amaranth‐based instant porridge had lower water‐binding potential (Daramola & Osanyinlusi, 2006). Low water‐binding potential is important for infant porridges as it maximizes flour rate and increases nutrient density. Peak time and pasting temperature were higher for amaranth‐based instant porridge than maize‐based instant porridge. Since a higher pasting temperature results from delayed swelling (Ho, Noor, & Bhat, 2012), it implies that amaranth‐based instant porridge is less viscous than maize‐based instant porridge. Amaranth‐based instant porridge is thus more desirable than the maize‐based porridge for feeding children under 5 years because of its low viscosity. Pasting temperature also provides an indication of the minimum temperature required to cook a given sample (Sandhu, Singh, & Malhi, 2007). The pasting temperature of amaranth‐based instant porridge was 94.75°C implying that water close to boiling is required to prepare the amaranth‐based instant porridge. The instant amaranth‐based porridge developed in this study can thus be easily prepared by mixing with hot water having a temperature ≥95°C without need for prolonged heating.

## 4 CONCLUSIONS

This study demonstrated that extrusion cooking can be used to develop acceptable and nutritious instant composite flours from locally available foods. It further revealed that ingredient complementation can be strategically applied in the development of extruded foods to boost sensory acceptability. Barrel temperature and feed moisture content do have varying effects on nutritional quality of extruded foods thus necessitating process optimization for products. Increasing barrel temperature and feed moisture content leads to a decrease in polyphenol, phytic acid contents, and iron extractability, but increases zinc extractability. Increasing feed moisture content favors retention of vitamin A. Optimal processing conditions for amaranth‐based porridge flours developed in this study were 14% feed moisture content and a barrel temperature of 169°C.

## CONFLICT OF INTEREST

None declared.
